# Prevalence of brain calcifications in a Brazilian cohort: A retrospective study in radiology services

**DOI:** 10.1590/S1980-57642013DN70200012

**Published:** 2013

**Authors:** Matheus Fernandes de Oliveira, Edison Barros e Silva, João Ricardo Mendes de Oliveira

**Affiliations:** 1 MD, Neurosurgery Residency Program. Hospital do Servidor Público Estadual de São Paulo, São Paulo SP, Brazil.; 2 MD, PhD, Neuropsychiatric Department - Federal University of Pernambuco (UFPE), Recife PE, Brazil.

**Keywords:** brain calcinosis, basal ganglia calcification, neuroimaging, neuroepidemiology

## Abstract

Brain calcifications may be present in a wide range of neuropsychiatric, infectious traumatic, neoplastic and endocrine conditions, associated with motor and cognitive symptoms. They are often considered "physiologic" or pathologic.

**OBJECTIVE:**

To determine the prevalence of brain calcifications in a Brazilian cohort.

**METHODS:**

1898 consecutive patients underwent skull CTs, comprising 836 men and 1062 women.

**RESULTS:**

Among all 1898 patients, 333 (197 women and 136 men) presented with brain calcifications, representing an overall prevalence of 17.54%. The prevalence in the female group was 18.54% versus 16.26% in the male group.

**CONCLUSIONS:**

A growth in incidental findings on CT scans are likely as these exams become more widely available. However, a current debate between radiologists and regulators is set to redefine the CT usage, especially in the United States and the United Kingdom, considering new norms for use and radiation dosage per exam.

## Abstract (pt)

Calcificações cerebrais podem estar presentes em uma grande variedade de condições médicas, incluindo doenças infecciosas, endocrinológicas, traumáticas, neoplásicas, psiquiátricas e neurológicas, associados a diferentes manifestações clínicas. Elas são muitas vezes consideradas "fisiológicas" ou patológicas.

**OBJETIVO:**

Determinar a prevalência de calcificações cerebrais em uma coorte brasileira.

**MÉTODOS:**

1898 pacientes consecutivos realizaram tomografia de crânio simples. 836 eram homens e 1062 eram mulheres.

**RESULTADOS:**

Entre todos os 1.898 pacientes, 333 (197 mulheres e 136 homens) apresentaram calcificações cerebrais. Assim, a prevalência total foi de 17,54%. No grupo feminino a prevalência foi de 18,54% e nos homens, 16,26%.

**CONCLUSÕES:**

Provavelmente os achados incidentais em tomografias computadorizadas (TC) serão crescentes, uma vez que estes exames estão mais disponíveis. No entanto, um debate atual entre radiologistas provavelmente deverá redefinir o uso de TC muito em breve, especialmente nos Estados Unidos e Reino Unido.

## INTRODUCTION

Brain calcifications may be present in a wide range of neuropsychiatric, infectious and endocrine conditions, associated with motor and cognitive symptoms.^1-9^.

These BCs are often considered "physiologic" or pathologic. Intracranial physiological calcifications are disconected of a demonstrable pathological cause and, especially in older subjects, are represented mainly by pineal gland, habenula and choroid plexus calcinosis. Other sites such as the vasculature or parenchyma are more often found in the context of clinical manifestations such as parkinsonism, dementia, psychosis and mood disorders.^1-11^

Physiological calcifications, such as in pineal gland, habenula and choroid plexus of lateral ventricles can appear in up to 50-70% of patients.^4-9^ Basal ganglia calcifications (BGC) are also well described in the context of idiopathic and secondary causes, having been demonstrated in various studies with a prevalence ranging from 0.3% to 12%.^1,11-14^

Brain calcinosis is usually found in patients over 30 years old, increasing progressively with age. Given the wider use of neuroimaging techniques, particularly tomography, BCs are increasingly viewed even in asymptomatic patients.^1,2,11-17^

Reports also depend on the profile of the health facility where data is gathered and analyzed because most can be highly biased towards a given level of severity, gender, age range or medical area of expertise such as Neurology, Psychiatry, Radiology or Geriatrics.

During a one-year period, 4219 consecutive computed tomograms (CT) were reviewed for basal ganglia calcifications and 14 patients harboring calcifications were identified. Calcifications on CT scans were bilateral in 12 cases and unilateral in 2 cases. The globus pallidus was the site of calcification in 13 of the 14 patients. Bilateral dentate nucleus calcification was seen in one patient.^1,18^

Ostling et al. (2003) studied the cross-sectional relationship between psychotic symptoms and BGC in a population sample of non-demented subjects, all of whom were 85 years old: 86 mentally healthy, 11 psychotic, 21 with mood disorders, and 20 subjects with anxiety disorders. Basal ganglia calcifications on CT were observed in 19% of the mentally healthy and 64% of the non-demented individuals with hallucinations or delusions. The authors concluded that basal ganglia calcification is strongly associated with psychotic symptoms in old age.^18^

Eskandary et al. (2005) found 3 cases of abnormal calcification, in the pineal region, basal ganglia, and temporal horn area, respectively, amongst 3000 CTs of head trauma patients with a mean age of 32±17.76 years attended at an emergency facility.^19^

Radaideh et al. (2012) studied a total of 1040 CT scans, observing an overall prevalence of basal ganglia calcifications of 1.25%. The prevalence increased with age; being 0.6% in younger age group vs. 2.4% in subjects older than 60 years. Elevated parathyroid hormone was found in 6 patients; of whom only one proved to have low vitamin D3 level.^20^

Calcifications in pediatric radiology became a much more common finding after the pandemic of children infected with HIV, most of whom acquired the condition from their infected mother.^1^

Few studies have specifically addressed the prevalence of other sites of brain calcifications and tend to associate their findings with specific symptoms. The objective of the present study was to determine the prevalence of brain calcifications in a Brazilian cohort.

## METHODS

A total of 1898 consecutive patients submitted to Skull computerized tomography (CT) had their CTs evaluated in order to determine the prevalence of intracranial calcifications. These images were consecutively performed at two different medical institutions (Hospital das Clínicas da Universidade Federal de Pernambuco e Instituto de Medicina Integral de Pernambuco) from April 2006 to April 2007. A total of 332 exams were collected at the Clinical Hospital of the Federal University of Pernambuco (HC-UFPE) and 1566 at the Instituto de Medicina Integral de Pernambuco (IMIP). This project was approved by the Research and Ethics Committee of the Federal University of Pernambuco.

All exams were evaluated by a single radiologist in order to determine the presence of pathological brain calcifications and their anatomical site (vascular and parenchymal). CTs were performed with a standardized protocol, consisting of slices of 3 mm in posterior fossa and 10 mm in the supratentorial space.

When evaluating age distribution, the WHO (World Health Organization) classification was employed, which considers the pediatric group as all patients under 20 years old; adults from 20 to 59 years old and elderly over 60 years old.

**Statistics.** The numerical data were expressed as mean ± standard deviation. The categorical data were expressed as percentages. Student's t-test was used for unpaired groups. The significance level was established as p<0.05.

## RESULTS

Overall, 1898 consecutive patients had their skull CTs evaluated. A total of 332 exams were conducted at the Hospital das Clínicas da Universidade Federal de Pernambuco (HC-UFPE) and 1566 at Instituto de Medicina Integral de Pernambuco (IMIP) involving 836 men and 1062 women. The mean age in the IMIP group was 34.25±24.82 years whereas the mean age in the HC-UFPE group was 47±23.27 years. There was a statistically significant difference between age profile in the two institutions (p<0.05).

In the IMIP group, of the 1556 patients, 689 were men and 877 were women. There was no statistical difference in age according to gender (p>0.05). In the HCUFPE group, of the 332 patients, 147 were men and 185 were women. There was no statistical difference in age between males and females (p>0,05).

**Figure 1:**
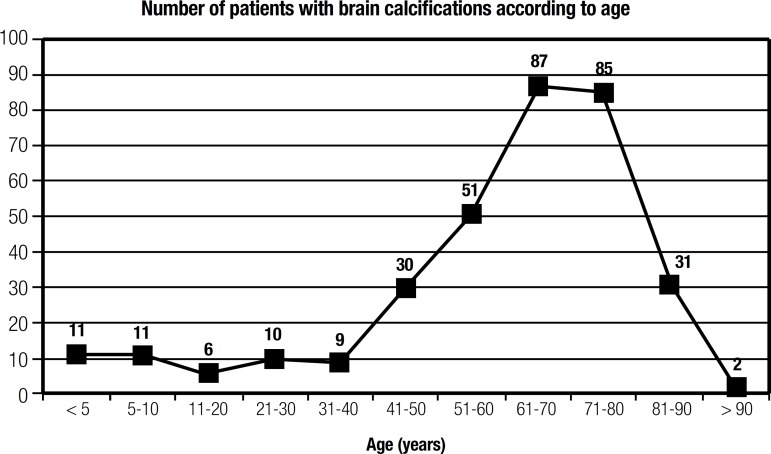
Number of patients with BC according to age group.

**Figure 2:**
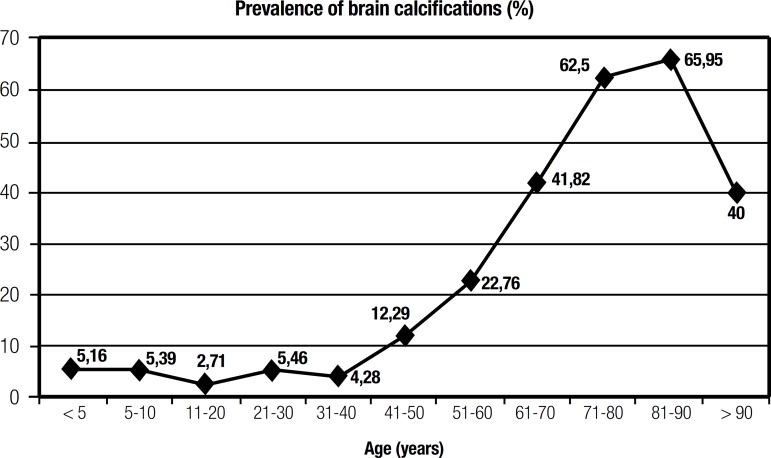
Prevalence of brain calcinosis across different age groups.

Among all 1898 patients, 333 (197 women and 136 men) presented with brain calcifications corresponding to an overall prevalence of 17.54%. The prevalence in the female group was 18.54% whereas among males the prevalence was 16.26%. The absolute number of patients with BC, and prevalence of BC according to age, are shown in Figures 1 and 2.

Among intracranial vascular calcifications, the carotid artery was the main site affected with a prevalence of 12.85%, followed by the vertebral artery at 1.84% and the basilar artery at 0.89%. There was no statistically significant difference between males and females.

Among parenchymal calcifications, the basal ganglia was the main site affected with a prevalence of 2.42%, followed by parietal at 1.36%, occipital at 0.52%, cerebral falx at 0.52%, frontal at 0.42%, cerebellum at 0.36%, and temporal at 0.26% of patients. Brainstem and cerebellar falx each accounted for 2 patients. Thalamus and sella represented 1 patient each (Table 1).

**Table 1:** Characteristics of brain calcifications.

Site		Patients	Prevalence	Mean age (years)	Age extremes	Gender predominance*
Vascular	Carotid	244	12.85%	66	1-102	F>M
	Vertebral	35	1.84%	68	1-93	F>M
	Basilar	17	0.89%	69	63-93	M>F
Parenchymal	Basal Ganglia	46	2.42%	50	1-88	F>M
	Parietal	26	1.36%	36	1-84	F>M
	Occipital	10	0.52%	36	7-79	F>M
	Cerebral Falx	10	0.52%	54	30-51	F>M
	Frontal	8	0.42%	38	14-68	F=M
	Cerebellar	7	0.36%	32	3-72	F>M
	Temporal	7	0.36%	41	11-68	F>M
	Brainstem	2	0.01%	45	40-50	F=M
	Cerebellar falx	2	0.01%	26	3-50	F=M

* Without statistical significance (p<0.05).

## DISCUSSION

Due to growing use of neuroimaging techniques, particularly CTs, brain calcifications are detected more often.^1,14^ Indeed, even the standardized protocol applied in most reference centers as well as in this study may underestimate the prevalence of calcifications. Multiple detector scanners are able to perform 0.5 mm thickness slices and thus calcifications smaller than this size may go undetected.

An intriguing consequence is the incidental finding of BC during the investigation of milder symptoms, some being transient, in asymptomatic subjects with massive calcifications and positive family history, in emergency rooms or even randomly during the investigation of other pathologies affecting the patient's face.^1,14^

For example, a pediatric case in which a 12-yearold girl presented transient symptoms who recovered completely after symptomatic treatment. A CT screening showed substantial calcinosis on her brain and also found in her other asymptomatic siblings.^21^ Other authors unexpectedly found three generations of asymptomatic carriers of brain calcinosis after investigating the kindred of a psychotic patient.^22^

Our analysis detected a prevalence of brain calcifications of 17.54% among 1898 CTs collected at two medical facilities, from subjects between 3 months of age and 103 years old. These two facilities had different profiles with one biased towards a younger population of children and the other focused on older adults and seniors. None of these facilities worked as emergency rooms and both focused equally on inpatient and outpatient caregiving. Intracranial vascular calcifications were the main findings, followed by basal ganglia calcifications. Although a female predominance was observed in most calcification types, statistical analysis failed to reveal a significant difference (p>0.05).

Interestingly, the hospital oriented for the adult group had higher rates of calcification. A significantly higher presence of basal ganglia calcification was detected in subjects over 60 years old, suggesting a probable link between calcinosis and the processes of aging and neuronal death. The prevalence of brain calcinosis across the different age groups is given in Figure 2 which also shows a progressive increase across different age groups when pooling together all sites of calcification detected, not only in basal ganglia but also including the cerebellum, white matter, pineal and vascular deposits.

Curiously, a dip in prevalence was observed after 90 years old. We hypothesized that there is a selection effect induced by calcification in the older old, with a smaller number of carriers after this cut-off, characterized by elder subjects with less prior calcinosis better brain health and consequently less calcification.

**Figure 3:**
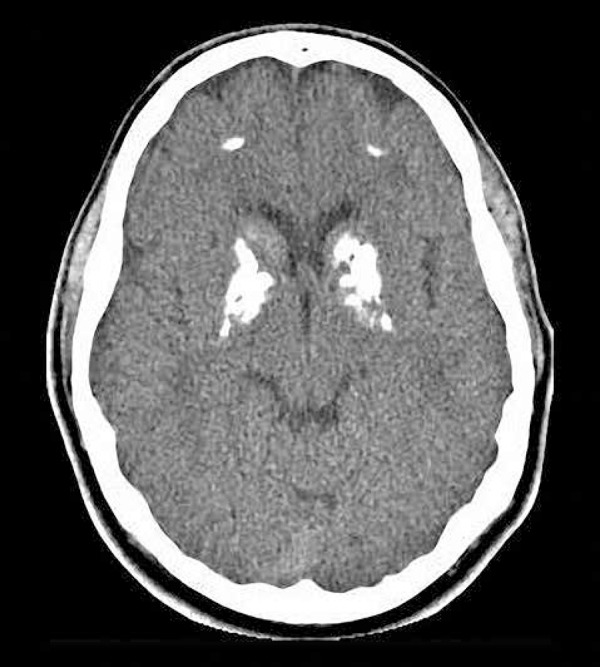
23 year-old subject with massive brain calcinosis, but only with mild symptoms, suggesting a high level of resilience for brain calcinosis.

We also identified unusual images from subjects with massive brain, calcinosis, yet only mild symptoms, suggesting a high level of resilience against brain calcinosis (Figure 3).

Intracranial vascular calcifications are usually associated with higher cardiovascular and ischemic cerebrovascular disease risk and may even represent a sign in the context of the atherosclerosis process.^23-25^ In children, vascular calcifications are also associated with obesity and cardiovascular diseases while chronic renal failure must also be excluded, since parathormone disturbances lead to ectopic calcifications.^26,27,28^

Parenchymal calcifications, on the other hand, represent a challenge, given they are found in a myriad of situations. Moreover, other sites of brain calcifications have also become the focus of recent attention, no longer merely as an incidental finding but as a predictive marker of prognosis in other neurodegenerative conditions. Using computed tomography, Mahlberg et al. (2008) found that the degree of pineal calcification in patients with AD was significantly higher than in patients with other types of dementia, depression or among controls.^29^ Another interesting fact is the already known association of pineal calcifications and psychotic syndromes, such as schizophrenia.^30^ Thus, mineralization remains a challenge in modern neuroscience, testing cerebral resilience and promoting the discussion over differences between physiological and pathological conditions.

In the reported sample, very few calcifications were depicted in the pineal gland, choroid plexus and habenula and this underreporting reveals the potential bias concerning conditions considered physiological or pathological. Although pathological calcifications are duly highlighted in radiological reports, physiological calcifications are sometimes not even included, based on the common sense premise that such findings do not represent harmful events and thus do not need further characterization. Considering the studied population and current scientific data, these calcifications would have involved up to 70% of our patients, well above the 0.02% encountered.

Undoubtedly, this unexpected finding may guide future discussions towards standardization of radiological protocols since a growth of incidental findings on CT scans are likely, as they become more widely available. Following the growing suspicion that some of the previously reported physiological calcifications may indeed have been pathological, many important observations and diagnosis are not being adequately conducted. Additionally, a current debate between radiologists and regulators is set to redefine CT usage, especially in the United States and the United Kingdom, considering new norms for use and radiation dosage per exam.^31^

In conclusion, brain calcifications are progressively more diagnosed and cited in the literature and new aspects about their clinical significance and implications are becoming clearer. There is an established association with advancing age and some of the previously considered physiological calcifications may indeed represent aspects of different nosologic conditions, thus stimulating discussion and standardization of radiological protocols.

While a thorough comprehension of patient features remains paramount, further basic science and clinical reports are expected to elucidate many unanswered questions.
